# One brain, one mind: A joint EPA–EAN leadership perspective on brain health

**DOI:** 10.1192/j.eurpsy.2026.12222

**Published:** 2026-05-25

**Authors:** Indrit Bègue, Pavel Mohr, Claudio L.A. Bassetti, Andrea Fiorillo, Paul A.J.M. Boon, Elena Moro, Geert Dom

**Affiliations:** 1 Laboratory for Neuroimaging and Translational Psychiatry, Department of Psychiatry, University of Geneva, Switzerland; 2 Adult Psychiatry Division, Department of Psychiatry, University Hospitals of Geneva, Switzerland; 3 Synapsy Center for Neuroscience and Mental Health Research, University of Geneva, Switzerland; 4 National Institute of Mental Health, Klecany, Czech Republic; 5 3rd Faculty of Medicine, Charles University in Prague, Czech Republic; 6 Medical Faculty University of Bern, Switzerland; 7 Neurology Department, Inselspital, Bern, Switzerland; 8 Department of Psychiatry,University of Campania “Luigi Vanvitelli”, Naples, Italy; 9 Ghent University (4Brain) & Ghent University Hospital (Neurology), Belgium; 10 Eindhoven University of Technology, Eindhoven, The Netherlands; 11 Grenoble Alpes University, Division of Neurology, CHU of Grenoble, Grenoble Institute of Neurosciences, INSERM U1216, Grenoble, France; 12 Collaborative Antwerp Psychiatric Research Institute (CAPRI), University of Antwerp, Belgium

**Keywords:** Brain Health, Mental Health, Psychiatry, Neurology

## Abstract

Neurology and psychiatry have operated as separate disciplines for over a century, yet this division reflects historical and institutional developments rather than the underlying biology of the brain. Contemporary neuroscience shows that brain and mental health disorders share genetic susceptibilities, inflammatory and metabolic pathways, environmental and social risk factors, and clinical features that cross diagnostic boundaries. Cognitive, emotional, sensory, and motor symptoms regularly appear across both neurological and psychiatric populations, and conditions such as seizures, psychosis, mood disorders, cognitive disorders, and sleep disorders are common to both. A brain health framework addresses this reality by treating the brain as a single biological organ whose function emerges from the interplay between genome and exposome – including stress, trauma, social context, existential meaning, pollution, and physical health – and which underlies perception, behaviour, cognition, emotion, resilience, and vulnerability. Translating this perspective into practice requires coordinated action across domains. Clinically, collaborative models such as joint neurology–psychiatry consultations and shared outpatient pathways can be implemented within existing resources to improve diagnostic clarity and continuity of care. In training, a more harmonised curriculum with shared foundations in neurobiology, joint seminars, and cross-rotations would equip clinicians with a common language while preserving specialist depth, and support the emerging fields of preventive neurology and preventive psychiatry. In research, organising studies around shared mechanisms and symptom dimensions, and launching joint funding calls, would enhance translational relevance and reduce duplication. To realise this vision, sustained leadership from European professional bodies is essential to establish collaboration as a shared professional standard.

The human brain does not recognise the boundaries we have constructed between neurology and psychiatry over the years. Yet these boundaries continue to define and organise much of our clinical practice, research design, and training. Their persistence reflects historical pathways rather than biological and neuroscientific principles, whereas the growing body of evidence clearly indicates shared mechanisms of brain and mental health disorders and calls for a more integrated framework [1, 2]. Bringing closer together the fields of neurology and psychiatry into a brain health framework does not entail merging established specialties, but ensures that their respective strengths contribute to a coherent and comprehensive approach to brain health [3].

The brain health framework recognises that the brain is a biological organ that is essential for human psychological, behavioural, physiological, and somatic processes. It enables individuals to experience their bodies, relationships, and environments. Psychiatric and neurological disorders emerge or evolve in interaction between genetic factors (genome) and environmental factors (exposome) such as stress, trauma, social context, existential meaning, air pollution, overall physical health, and others. As such, a comprehensive brain health approach explores the brain and these complex interactions in shaping perception, behaviour, cognition, emotions, resilience, and vulnerability. Indeed, physical, psychosocial, and existential factors leave measurable traces in brain function and structure, just as biological processes contribute to emotional, cognitive, and behavioural outcomes. Recognising this reciprocity does not dilute the scientific foundations of neurology or psychiatry. Rather, it broadens the clinical lens through which both fields understand illness and supports a more coherent, holistic, and unified model of brain health and recovery.

Historically, there was a single medical field dealing with nervous diseases, “neuropsychiatry.” The separation into two disciplines, neurology and psychiatry, started at the beginning of the twentieth century. While neurology focused on identifiable brain pathology (“physical brain”), psychiatry dealt with functional, cognitive, behavioural, and mood disorders (“mind”). The split was also driven by evolving disease classification systems, emerging psychological theories, and the increasing specialisation and institutional differentiation of medical practice. Over time, though, functional divisions intended to support education and service organisation acquired the weight of conceptual categories. Today, however, clinical experience and advances in neuroscience demonstrate the extent to which many brain and mental health disorders cross traditional boundaries between the specialties, supporting calls for closer integration [4, 5]. Individuals diagnosed with neurological conditions frequently present with cognitive or emotional changes that influence prognosis and quality of life. Patients treated within psychiatric services often show cognitive, sensory, or motor features that benefit from neurological expertise. Seizures, cognitive disorders, mood disorders, and sleep disorders are very prevalent in both neurological and psychiatric populations [6]. In addition, shared risk factors [7] and the frequent comorbidity between neurological and psychiatric disorders, their shared genetic susceptibilities, inflammatory pathways, metabolic factors, and environmental influences further underline the continuity of brain health across diagnostic groups, making the historical divide increasingly difficult to justify.

Therefore, the question is how best to adapt our systems, that is, clinical, education, and research, to this contemporary understanding of the brain. Collaborative clinical models (“brain health hubs”) are a pragmatic starting point because they address immediate needs without requiring major structural reforms. In many European centres, neurologists and psychiatrists already encounter patients whose presentations do not fall clearly within one discipline. When services are configured to allow timely joint consultations, shared outpatient sessions, or coordinated follow-up plans, clinicians can combine their perspectives early in the clinical trajectory rather than sequentially and in isolation. This approach reduces diagnostic uncertainty, avoids duplicate investigations, and provides patients with a clearer and more coherent explanation of their symptoms. It also supports clinicians, who often recognise that complementary expertise is needed but lack formal pathways to access it. These models can be implemented within existing resources and can significantly improve continuity of care. European professional leadership can guide and reinforce these developments by setting shared expectations for collaborative practice, promoting standards for integrated service pathways, and supporting centres that pilot joint neurology–psychiatry clinics.

Education and training are key areas where more alignment is needed [8]. Training programmes in neurology and psychiatry already include elements of the other discipline, but these components often function in parallel rather than in dialogue. However, both the quality and quantity of this cross-training differ widely between European countries. A more coordinated structure could provide a shared foundation in neurobiology, development, behaviour, and systemic influences on brain function, while still allowing each specialty to cultivate its distinctive depth. Joint seminars supervised cross-rotations, and regular case-based discussions would help trainees build a common clinical language and understand when collaborative input is essential. Such an interdisciplinary curriculum could also support public health strategies, and incorporate preventive neurology and preventive psychiatry, areas that sit uneasily within current structures, although they are central to long-term brain and mental health. Whether conceived as a shared educational platform or as the foundation for a future specialty, these activities would strengthen rather than blur specialist identity by giving clinicians a broader and more integrated understanding of the organ both fields serve. Clinical care for brain disorders should reflect this integrated perspective, drawing on the full range of evidence-based interventions. Innovations in pharmacology, neuroimaging, neuromodulation, digital therapeutics, virtual-reality-based rehabilitation, and AI-supported clinical tools provide new opportunities to address symptoms that span diagnostic neuropsychiatric categories. When neurologists and psychiatrists work within a coordinated model of care, each benefits from the other’s competencies and an increasingly shared toolbox of both disciplines, combining tools and approaches in a clinically meaningful way.

Research similarly benefits from integration, as illustrated by efforts, for example, in the field of behavioural disorders, epilepsy [9], schizophrenia, cognition/dementia [10], sleep disorders [6], functional disorders [11], headache, and others. Indeed, a better collaboration between neurology and psychiatry builds on the reality that brain and mental health disorders dominate the burden of disability globally [12]. The economic burden is equally substantial, with recent analyses demonstrating the enormous costs of brain and mental health disorders to healthcare systems and society [13]. Thus, brain health and related conditions should be a priority of health policies, supported by sufficient economic resources. Many of the most important questions in brain health, from early identification of risk to personalised intervention strategies, require complementary scientific approaches. Studies organised around shared mechanisms rather than historical diagnostic categories can better reflect the complexity of brain disorders and improve translational relevance. Joint funding calls across symptom dimensions across neurology and psychiatry, rather than diagnostic categories, would strongly encourage this type of collaboration. By inviting interdisciplinary teams to develop proposals from the outset, such initiatives would both broaden scientific scope and help consolidate methodological standards across the field. Coordinated research priorities at the European level could accelerate progress and reduce duplication, particularly in areas where resources are limited.

Professional leadership is essential to give coherence to these developments. The European Psychiatric Association and the European Academy of Neurology have already articulated overlapping commitments to improving brain health, workforce development, and evidence-based practice. Coordinated statements, shared guideline development where appropriate, and co-sponsored educational events would reinforce the message that collaboration is a professional expectation rather than an occasional exception. National societies often shape service organisation and training standards, and alignment at the European level can help set a clear direction for future development.

Taken together, these strategies point toward a more integrated vision of brain health that may benefit from the different strengths of neurology and psychiatry and recognise their deep scientific and clinical interdependence, as stressed recently by the Bern Declaration on Brain Health [14]. The goal is not to erase distinctions that have value, but to ensure that they do not impede patient care or scientific progress. As understanding of brain disorders advances and as patient needs become more complex, a system that facilitates collaboration rather than reinforces separation will better serve clinicians, researchers, and the public.

## References

[1] Fiorillo A. A roadmap for better and personalized mental health care in Europe: the priorities of the European psychiatric association. Eur Psychiatry. 2025;68:e60. 40405767 10.1192/j.eurpsy.2025.2456 PMC12188326

[2] Boon PAJM, Berger T, Leonardi M, Marson T, Kallweit U, Moro E, et al. A roadmap toward promoting and improving brain health in Europe and closing the awareness and funding gap. Eur J Neurol. 2025;32:e16589. 39815708 10.1111/ene.16589 PMC11735729

[3] Lakhan SE. Abolish psychiatry, abolish neurology: toward a unified discipline of brain medicine. Cureus. 2025;17:e84817. 40420967 10.7759/cureus.84817 PMC12105051

[4] Fitzgerald M. Do psychiatry and neurology need a close partnership or a merger? BJPsych Bull. 2015;39:105–7. 26191446 10.1192/pb.bp.113.046227 PMC4478930

[5] Perez DL, Keshavan MS, Scharf JM, Baslet G, Dickerson BC, Rosenbaum JF, et al. Bridging the great divide: what can neurology learn from psychiatry? J Neuropsychiatry Clin Neurosci. 2018;30:271–8. 29939105 10.1176/appi.neuropsych.17100200 PMC6309772

[6] Bassetti CL, Ferini-Strambi L, Brown S, Adamantidis A, Benedetti F, Bruni O, et al. Neurology and psychiatry: waking up to opportunities of sleep. : State of the art and clinical/research priorities for the next decade. Eur J Neurol. 2015;22:1337–54. 26255640 10.1111/ene.12781

[7] Senff J, Tack RWP, Mallick A, Smith L, Gimson A, Llewellyn DJ, et al. Modifiable risk factors for stroke, dementia and late-life depression: a systematic review and DALY-weighted risk factors for a composite outcome. J Neurol Neurosurg Psychiatry. 2025;96:515–27. 40180437 10.1136/jnnp-2024-334925

[8] Brown JC, Dainton-Howard H, Woodward J, Cavanagh J, David AS, Espay AJ, et al. Time for brain medicine. J Neuropsychiatry Clin Neurosci. 2023;35:333–40. 37021384 10.1176/appi.neuropsych.21120312

[9] De Giorgi R, Lomax T, Moreno C, Arango C, Bassetti CLA, Cavanagh J, et al. Epilepsy and mental health disorders: current challenges and potential solutions. Br J Psychiatry J Ment Sci. 2025;1–8. 10.1192/bjp.2025.10455 41261735

[10] Livingston G, Huntley J, Liu KY, Costafreda SG, Selbæk G, Alladi S, et al. Dementia prevention, intervention, and care: 2024 report of the lancet standing commission. Lancet. 2024;404:572–628. 39096926 10.1016/S0140-6736(24)01296-0

[11] Bègue I, Nicholson TR, Kozlowska K, Aybek S, Carson A, David AS, et al. Psychiatry’s modern role in functional neurological disorder: join the renaissance. Psychol Med. 2021;51:1961–3. 34167595 10.1017/S0033291721002038

[12] GBD 2021 Diseases and Injuries Collaborators. Global incidence, prevalence, years lived with disability (YLDs), disability-adjusted life-years (DALYs), and healthy life expectancy (HALE) for 371 diseases and injuries in 204 countries and territories and 811 subnational locations, 1990-2021: a systematic analysis for the global burden of disease study 2021. Lancet Lond Engl. 2024;403(10440):2133–61. 10.1016/S0140-6736(24)00757-8 PMC11122111 38642570

[13] Mitchell AJ, Cogswell IE, Dalos J, Nichols E, Whiteford HA, Vigo D. Estimating global direct health-care spending on neurological and mental health between 2000 and 2019: a modelling study. Lancet Public Health. 2025;10:e401–11. 40312084 10.1016/S2468-2667(25)00089-1

[14] Bassetti CLA, Bègue I, Chen C, Espay AJ, Husain M, Menon DK, et al. The Bern declaration on brain health: a decalogue to launch an international alliance. Lancet Neurol. 2025;24:720. 40818468 10.1016/S1474-4422(25)00286-8

